# Citrullination in tumor immunity and therapy

**DOI:** 10.1172/JCI196348

**Published:** 2025-10-15

**Authors:** Michael R. Pitter, Weiping Zou

**Affiliations:** 1 Department of Surgery, University of Michigan Medical School, Ann Arbor, Michigan, USA.; 2 Center of Excellence for Cancer Immunology and Immunotherapy, University of Michigan Rogel Cancer Center, Ann Arbor, Michigan, USA.; 3 Graduate Program in Immunology, University of Michigan, Ann Arbor, Michigan, USA.; 4 Department of Pathology, University of Michigan Medical School, Ann Arbor, Michigan, USA.; 5 Graduate Program in Cancer Biology and; 6 University of Michigan Rogel Cancer Center, University of Michigan, Ann Arbor, Michigan, USA.

## Abstract

Peptidyl arginine deiminases (PADs) catalyze the conversion of arginine residues into peptidyl citrulline, a posttranslational modification known as protein citrullination (or arginine deimination). This process alters the charge of proteins from positive to neutral, thereby affecting their folding, stability, conformation, and function. PAD2 and PAD4 can translocate into the nucleus and citrullinate both cytoplasmic and nuclear proteins. In this Review, we focus on PAD2- and PAD4-mediated citrullination in immune cell subsets within the tumor microenvironment. We discuss how citrullination regulates immune cell function and tumor immunity and explore the potential of targeting citrullination as a strategy for cancer immunotherapy.

## Introduction

**Figure 1:**
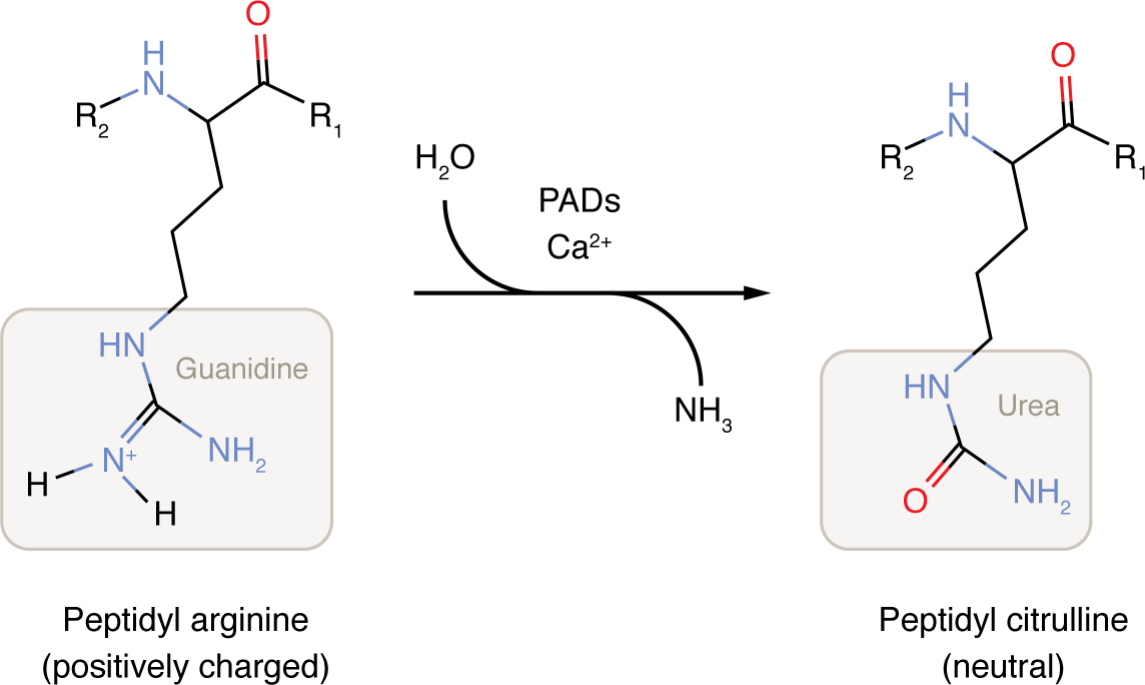
Scheme of PAD-mediated citrullination. PADs catalyze the conversion of arginine residues into peptidyl citrulline. This process alters the charge of proteins from positive to neutral, thereby affecting protein folding, stability, conformation, and thereby function.

The genome gives rise to the transcriptome, which, in turn, gives rise to the proteome (1, 2). Posttranslational modifications (PTMs) shape final protein functions (3–8). PTMs influence protein folding, stability, and three-dimensional conformation (9–11). These modifications include the covalent addition of chemical groups (such as phosphorylation, methylation, acylation, and acetylation); the addition of large unique molecules (such as ADP-ribosylation, prenylation, and glycosylation); the addition of polypeptides (such as ubiquitination and SUMOylation); proteolytic cleavage; and the modification of amino acids (such as glycation, oxidation, carbamylation, and citrullination) (12, 13). One key distinction among PTMs is their reversibility (12, 13). Most PTMs are reversible, while others — such as proteolysis and citrullination — are irreversible (3, 12, 14–17). Proteolysis, though common, often results in protein inactivation (18, 19), whereas citrullination is the only known irreversible enzymatic PTM that modifies amino acid identity by converting arginine into citrulline (13, 20). The discovery of citrullinated proteins dates to 1958, when G.E. Rogers and colleagues reported the presence of citrulline in fibrous proteins of rat hair follicles (21), building on earlier observations in red algae (22). The identification of citrulline in human brain myelin proteins in 1971 marked a turning point in citrullination research (23, 24). Citrullination involves peptidyl arginine deiminase–catalyzed (PAD-catalyzed) hydrolysis of the guanidino group in peptidyl arginine, converting it into urea and releasing ammonia, thereby forming peptidyl citrulline (25, 26) (Figure 1). This modification alters a protein’s structure and function (3, 27–31). Later studies revealed that PAD2 and PAD4 citrullinate histones, affecting chromatin structure and gene transcription (32–36). Histone citrullination can promote neutrophil extracellular trap (NET) formation, DNA damage responses, and transcriptional regulation (32, 34). However, the roles of PAD2 and PAD4 in nonhistone protein citrullination within the tumor microenvironment (TME) remain largely unexplored. This Review highlights citrullination in immune cells and its implications for tumor immunity and therapy.

## PAD family member expression and distribution

The PAD family of isozymes — PAD1, PAD2, PAD3, PAD4, and PAD6 — catalyzes protein citrullination (25, 26) (Figure 1). PAD1 is predominantly expressed in the epidermis, hair follicles, uterus, and stomach; PAD3 in hair follicles, skin, and peripheral nerves; and PAD6 in reproductive tissues, including ovaries, oocytes, embryos, and testes (25, 37). Notably, PAD6 lacks several conserved Ca²^+^-binding residues required for enzymatic activity (38), and its catalytic function has not been demonstrated (39, 40).

PAD2 exhibits broad tissue distribution, including skeletal muscle, salivary glands, brain, skin, peripheral nerves, uterus, pancreas, kidney, and inner ear. PAD4 is more restricted, primarily expressed in the brain and uterus. Importantly, PAD2 and PAD4 are the major PAD isozymes found in immune cells (25, 35).

PAD4 uniquely possesses a canonical nuclear localization signal (NLS), enabling its translocation to the nucleus, where it citrullinates histones — an essential step in NET formation in neutrophils (32, 33, 41). Although PAD2 lacks a canonical NLS, it can also localize to nucleus and modify nuclear substrates (35, 42, 43). Histone citrullination by PADs can influence chromatin architecture and epigenetically regulate transcription (33, 34, 44).

**Figure 2:**
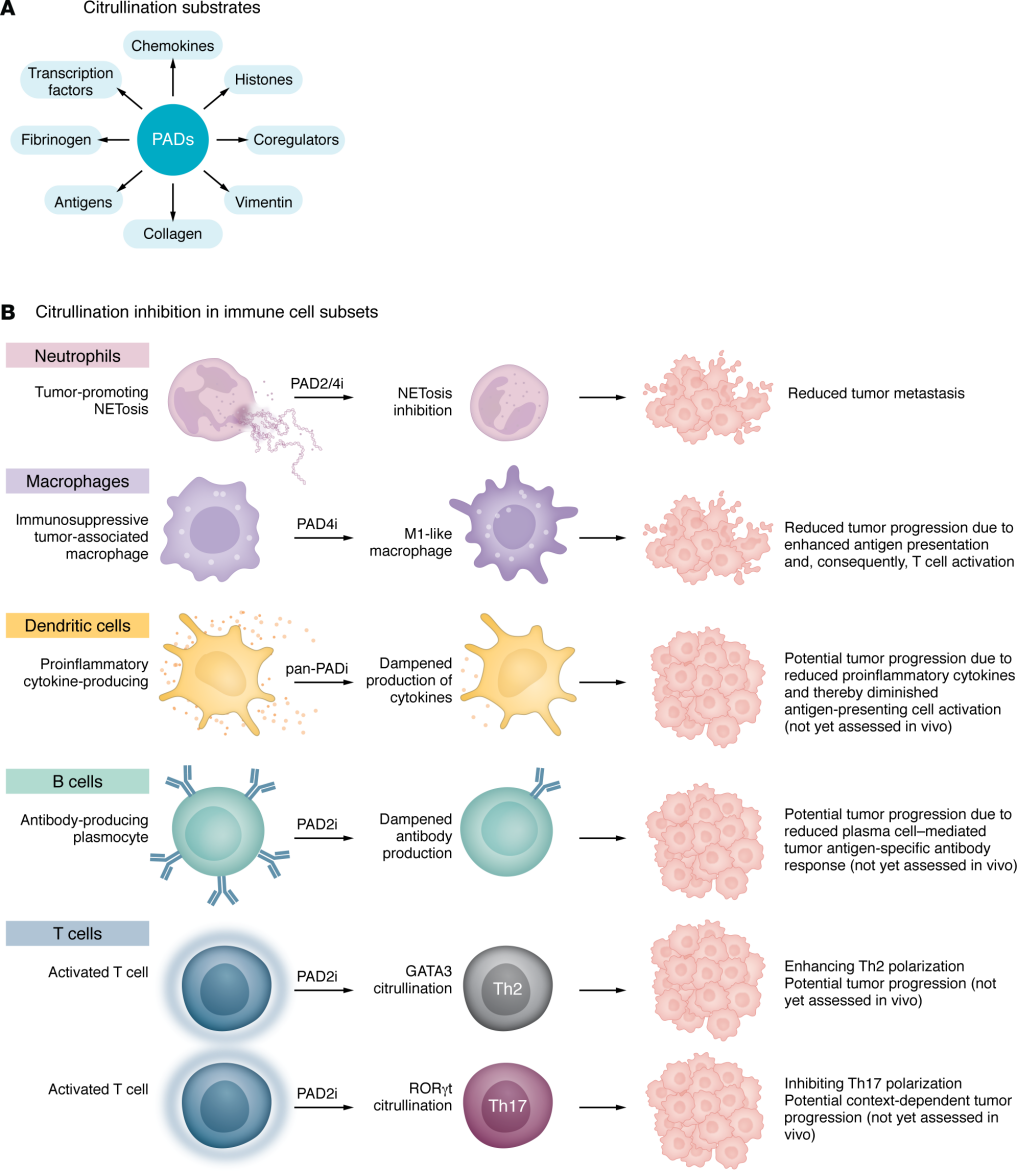
PAD-mediated citrullination. (**A**) Citrullination substrates. (**B**) Citrullination inhibition in immune cell populations and potential biological outcomes.

The functional consequences of PAD-mediated citrullination are determined by their targeted substrates. PAD2 and PAD4 target various cytoplasmic and nuclear proteins, including transcription factors and coregulators (45, 46) (Figure 2A). Their expression in hematopoietic progenitors and immune cells and their ability to enter the nucleus suggest important roles in modulating immune cell differentiation, phenotype, and function (Figure 2B) (47).

## Citrullination in immune cells and tumor immunity

Given that citrullination occurs in immune cells, it is expected to influence tumor immune responses. Here, we review both the established and emerging roles of PAD-mediated citrullination across various immune cell subsets within the TME.

### Citrullination in neutrophils.

Most studies of PADs in neutrophils have focused on autoimmune conditions. Inflammatory stimuli, including ROS, TLR agonists, and proinflammatory cytokines can drive intracellular Ca²^+^ influx and activate PAD4, leading to histone citrullination, chromatin decondensation, and NET formation (48–51). Additionally, PAD4 citrullinates p65 in response to LPS stimulation, thereby inducing transcription of inflammatory cytokines, such as IL-1β and TNF-α (52). Cancer cells can exploit NETs to promote metastasis (53, 54). Chemotherapy-induced NET formation also contributes to immune evasion and resistance via TGF-β signaling (55–58). NETs can reactivate dormant tumor cells and promote metastatic outgrowth (59). Inhibiting PAD4 or degrading chromatin with DNase I have been shown to reduce tumor metastasis and prolong survival in tumor-bearing models (60). PAD2 also contributes to NET formation by citrullinating histones (61). Thus, PADs in neutrophils may promote a proinflammatory milieu, facilitating tumor progression.

Notably, there may exist a distinct antitumorigenic neutrophil subset, positively correlating with immune checkpoint blockade (ICB) efficacy (62). In line with this, NETs induced by chemotherapy have been shown to release tumor-killing granules, containing elastase, myeloperoxidase, and cathepsin G, in a murine colon cancer model (63). It remains to be studied how PADs affect different neutrophil subsets and ICB in patients with cancer.

### Citrullination in macrophages.

**Figure 3:**
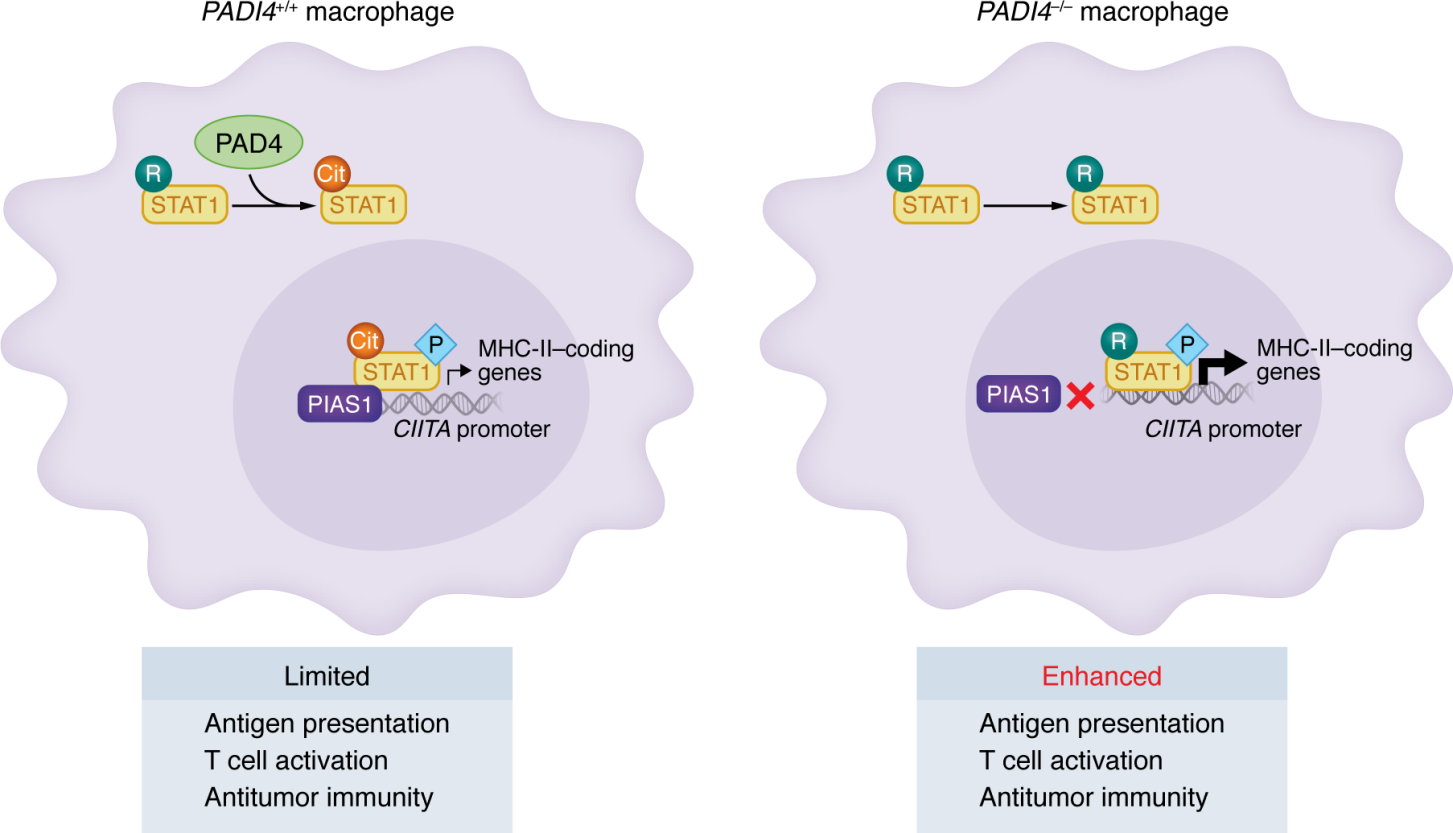
The relationship between PAD4 and MHC-II expression in macrophages. PAD4 mediates citrullination of STAT1 in tumor-associated macrophages (TAMs). This results in enhanced interaction between STAT1 and PIAS1, thereby reducing STAT1 binding to the CIITA gene promoter, downregulating MHC-II expression, and consequently impairing tumor immunity. The opposite phenotype is shown in *PAD4^–/–^* (*PAD4-*deficient) macrophages.

Tumor-associated macrophages (TAMs) can play an immunosuppressive role in antitumor immune responses via distinct mechanisms (64). For example, antigen-presenting cells, including macrophages and DCs, express high levels of PD-L1 (also known as B7-H1) in the human TME and tumor-draining lymph nodes, contributing to immune resistance to ICB in the TME (65–68). Interestingly, PAD4 is highly expressed in macrophages (47, 69), including TAMs (70). It is not clear if PD-L1 and PAD4 are differentially expressed in TAMs. PAD4 citrullinates STAT1 in TAMs, enhancing the interaction between STAT1 and protein inhibitor of activated STAT1 (PIAS1) and suppressing STAT1 transcriptional activity (70). This attenuates class II transactivator (CIITA) transcription and reduces MHC-II expression in TAMs, thereby impairing antigen presentation and T cell activation (70–72) (Figure 3). PAD2 and PAD4 also regulate inflammasome activation and pyroptosis in macrophages. For example, PAD2 citrullinates apoptosis-associated speck-like protein containing a CARD (ASC) specks, leading to caspase-1 activation and IL-1β and IL-18 release (73, 74). Inhibition of PAD4 with the small-molecule inhibitor GSK484 reduces IL-1β secretion following NOD-like receptor family pyrin domain containing 3 (NLRP3) activation. PAD2-specific inhibition with AFM30a more effectively diminishes IL-1β production in PAD4-deficient macrophages, suggesting functional redundancy and synergy of PAD2 and PAD4 (73). PAD2 knockout reduces pyroptosis in bone marrow–derived macrophages and alveolar macrophages (74). Thus, PAD2 and PAD4 generally support inflammatory cytokine production in macrophages.

Moreover, like neutrophils, macrophages can also form extracellular traps (METs). PAD2 and PAD4 promote MET formation in autoimmune models (75, 76). METs can exacerbate or alleviate tumor progression depending on the context (77–81). While inflammatory cytokines can target tumor cells and stromal cells to promote tumor growth, they can also stimulate antigen-presenting cells, enhancing their antigen presentation and T cell priming and activation. Thus, PADs in macrophages may play dual roles in tumor immunity depending on their cellular targets and immune context in the TME. Given that treatment with PAD4 inhibitors (GSK484 and JBI-589) reduces tumor progression and enhances therapeutic efficacy of ICB in different tumor-bearing mouse models (70, 82), inhibition of PAD4 is a potential strategy for cancer immunotherapy.

### Citrullination in T cells.

Citrullination is relatively understudied in T cells. A seminal study showed that PAD2 can citrullinate the transcription factors GATA-binding protein 3 (GATA-3) and retinoic acid–related orphan receptor γ, thymus-specific isoform (RORγt), thereby modulating Th cell polarization. Specifically, PAD2-mediated citrullination of GATA-3 reduced IL-13 promoter activity, whereas citrullination of RORγt enhanced IL-17A promoter activity, thereby promoting Th17 differentiation (83). Structural analysis revealed that citrullination of arginine 330 (R330) on GATA-3 — a residue that interacts with the DNA backbone — disrupted its DNA binding and dampened Th2 polarization. In contrast, citrullination of RORγt enhanced its DNA binding and transcriptional activity, favoring Th17 lineage commitment (83). Although this study was conducted in an inflammatory setting, it suggests that PAD2 may influence CD4^+^ T cell polarization within the TME. Another study demonstrated that PAD2 and/or PAD4 deficiency impairs both Th1 and Th17 cell differentiation in a TLR7-driven lupus model (84). Given that Th1 and Th17 cells play important roles in antitumor immunity (85–88), these findings raise the possibility that PADs modulate effector T cell responses in the TME.

Moreover, PAD2 can citrullinate chemokines such as CXCL10 and CXCL11 — key mediators of T cell trafficking — thereby impairing T cell migration to inflammatory sites, including tumors (89, 90). Although this mechanism has not yet been explored in the context of cancer, altered chemokine citrullination could influence effector T cell trafficking into the TME.

In autoimmune settings, such as rheumatoid arthritis (RA), both CD4^+^ and CD8^+^ T cells recognize citrullinated self-antigens, including vimentin, fibrinogen, and enolase (91–93). Analogously, whether T cell responses are triggered by citrullinated tumor or stromal proteins in cancer remains unknown. Altogether, these findings suggest that citrullination may shape T cell phenotype, tumor migration, and the antigenic landscape of tumors, thereby impacting T cell–mediated tumor immunity.

### Citrullination in other immune cells.

In addition to neutrophils, macrophages, and T cells, citrullination has also been studied in B cells and DCs (94). PAD2 citrullinates marginal zone B and B1 cell–specific protein (MZB1) at multiple sites, modulating B cell activation, as well as antibody production and secretion (95). Pharmacological inhibition of PAD2 with AFM30a significantly reduced IgA and IgM levels in human plasmablasts, while IgG levels remained unaffected (95). Like T cells in RA, autoreactive B cells can respond to citrullinated proteins — such as collagen type II — in the synovial fluid, further exacerbating inflammation in patients with RA (92).

In DCs, PAD inhibition alters cytokine production and functional maturation. For instance, the pan-PAD inhibitor Cl-amidine reduces DC secretion of TNF-α, IL-6, IL-1β, and IL-12p70 in response to various TLR agonists (96). Consistent with this, Cl-amidine also downregulates inducible nitric oxide synthase (iNOS) expression in DCs (97). Notably, iNOS deficiency has been associated with enhanced tumor progression in melanoma models (98, 99). PADs also regulate the transdifferentiation of DCs into osteoclasts. Krishnamurthy et al. found that PAD2 and PAD4 are expressed during this process — with PAD2 predominantly in the cytoplasm and PAD4 in the nucleus — and that multiple substrates, including actin and vimentin, are citrullinated before, during, and after this transition (100). Inhibition with Cl-amidine significantly impaired osteoclast differentiation from immature DCs. Given that tumors, such as prostate and breast cancer, frequently metastasize to the bone marrow, it will be important to investigate whether PAD activity contributes to or modulates tumor bone marrow metastasis. Together, these findings suggest that PAD2 and PAD4 regulate B cell and DC biology in both physiological and pathological conditions, with potential implications for shaping tumor immune responses and therapeutic outcomes.

## Potential relationship between arginine methylation and citrullination

Arginine methylation and citrullination often act as functionally antagonistic modifications (101, 102). For example, STAT1 arginine methylation enhances its transcriptional activity in IFN-stimulated U266B1, 2fTGH, and HEK 293T cell lines (103), whereas PAD4-mediated citrullination of STAT1 promotes its association with PIAS1, thereby reducing STAT1 binding to CIITA promoter regions and impairing MHC-II expression in macrophages (70).

Emerging evidence suggests that arginine methylation is essential for antitumor immunity. For example, protein arginine methyltransferase 5 (PRMT5) can mediate methylation of Sm proteins in T cells. This supports γc-family cytokine signaling by facilitating the splicing of Il2rg and Jak3 transcripts, leading to proper expression of IL-2 receptor and Janus kinase 3 (JAK3) (104). This observation raises the possibility that PAD-mediated citrullination could antagonize γc-cytokine signaling, thereby impairing T cell responses to tumors. Similarly, methylation of the nuclear factor of activated T cells (NF-AT) coactivator NF-AT–interacting protein 45 (NIP45, also known as ILF2BP) is required for IFN-γ production in T cells, and its inhibition reduces T cell cytokine expression (105). Thus, citrullination and methylation may compete for the same arginine residues on immune regulatory proteins, shaping immune cell function in the TME (106–111). Comprehensive proteomic profiling of methylated and citrullinated proteins in immune cells will help identify novel PAD substrates and elucidate the interplay between these competing PTMs in modulating tumor immunity (26, 40, 112).

## Therapeutically targeting citrullination

PAD inhibitors were initially developed for treating autoimmune diseases such as RA (113–117). More recently, these inhibitors have been applied to target citrullination in cancer cells and tumor-infiltrating neutrophils (118) (Table 1).

PAD inhibitors include both pan-PAD inhibitors and those selective for individual isozymes. Activity-based protein profiling high-throughput screening has identified reversible and irreversible inhibitors. Reversible inhibitors such as minocycline, chlortetracycline, ruthenium red, and sanguinarine typically act by chelating calcium, which is essential for PAD activation (119–122). Irreversible PAD inhibitors can covalently bind to the active-site cysteine of PADs, directly inhibiting enzymatic activity (114, 123, 124). Other irreversible PAD inhibitors such as NSC95397 and streptonigrin possess α,β-unsaturated carbonyl groups that covalently modify active-site cysteines (114). Streptonigrin shows high PAD4 selectivity and potency due to its substituted pyridyl and phenyl rings (125). GSK121 is a selective PAD4 inhibitor, and its further chemical optimization yielded GSK199 and GSK484, which exhibit enhanced potency and selectivity (126). 2-chloroacetamidine derivatives broadly inhibit PADs but show higher potency against PAD2 and PAD4. Cl-amidine, BB-Cl-amidine, and F-amidine are such derivatives; F-amidine and GSK484 are PAD4 selective (126–130). YW3-56, a Cl-amidine derivative, inhibits PAD1-4 with improved potency (131, 132). BMS-P5 and JBI-589 are next-generation PAD4 inhibitors that suppress NET formation and neutrophil recruitment, mitigating tumor progression in preclinical models (82, 133). Notably, BB-Cl-amidine may also act as a STING inhibitor and impair innate tumor immune responses (134). Additionally, GSK484 and JBI-589 can inhibit PAD4 by binding the *calcium-free* form of the isozyme and, therefore, may potentially be disengaged by high levels of calcium, particularly in the extracellular microenvironment (135, 136).

PAD2-selective inhibitors include benzimidazole-based compounds such as AFM30a, AFM32a, AFM41a, and AFM49a. These compounds have shown efficacy in preclinical models of PAD2-driven sepsis, endotoxic shock, and thrombosis (137–140). Recent studies using phage display have identified monoclonal antibodies that selectively activate or inhibit extracellular PAD4 activity, offering additional therapeutic avenues (141).

Overall, PAD inhibitors represent a promising class of therapeutic agents with potential application in cancer (Table 1). By disrupting PAD-mediated posttranslational and epigenetic modifications, these agents may modulate immune cell function and suppress tumor-promoting inflammation.

## Conclusion

PTMs, including citrullination, are fundamental in regulating the structure, stability, and function of proteins. Citrullination modulates transcriptional programs in cancer cells and immune cells by altering protein conformation and protein-protein interactions (35, 142). In neutrophils, PAD4-mediated histone citrullination facilitates NET formation, which can enhance tumor progression and metastasis (54, 55). In macrophages, PAD-dependent citrullination regulates antigen presentation and inflammatory cytokine production (70). Despite these insights, the roles of PAD2 and PAD4 in other immune subsets, including T cells, B cells, and DCs, remain understudied. Notably, PAD4 directly citrullinates and activates NF-κB in neutrophils (52), indicating that there is an opportunity to explore the roles of NF-κB citrullination in other innate and adaptive immune cells whose effector functions are governed by NF-κB signaling.

Recent preclinical studies demonstrate that PAD inhibition can suppress tumor growth and metastasis, indicating a therapeutic potential in oncology (70, 82, 133). These findings underscore the need for a deeper mechanistic understanding of PAD isozyme activity within specific immune cell populations in the TME. Development of isozyme-selective inhibitors and novel PAD-targeting strategies could offer new immunotherapeutic avenues. Nevertheless, since the inhibition of PAD2 and PAD4 in tumor cells has shown antitumor efficacy in animal models (70, 82), targeting PAD activity in both immune and tumor cells may produce synergistic anticancer effects. For instance, PAD4 inhibition in TAMs enhances antigen presentation and T cell activation, and a similar mechanism in tumor cells could further promote tumor-specific T cell responses. Future research should focus on identifying citrullinated substrates in immune cells, dissecting PAD-mediated regulatory pathways, and evaluating PAD-targeted therapies in combination with different therapies, including ICB. By advancing our understanding of citrullination in cancer immunity, we may uncover novel opportunities for enhancing the efficacy of cancer immunotherapy.

## Figures and Tables

**Table 1:**
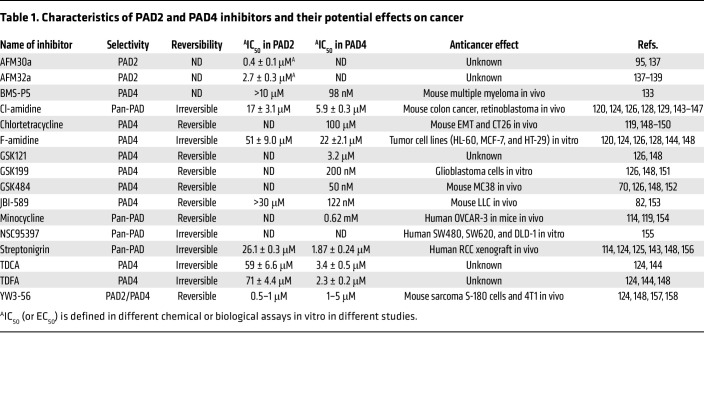
Characteristics of PAD2 and PAD4 inhibitors and their potential effects on cancer

## Version 1 10/15/2025

Electronic publication
